# A meta-analysis on randomized controlled trials of treating eosinophilic esophagitis with budesonide

**DOI:** 10.1080/07853890.2022.2101689

**Published:** 2022-07-21

**Authors:** Xiaopei Liu, Xue Xiao, Dan Liu, Cong’e Tan

**Affiliations:** a School of Basic Medicine, Shaanxi University of Chinese Medicine, Xianyang, Shaanxi, China; b Department of Anorectal, Xi’an Hospital of Traditional Chinese Medicine, Xi’an, Shaanxi, China

**Keywords:** Budesonide, eosinophilic esophagitis, meta-analysis

## Abstract

**Objective:**

Eosinophilic esophagitis (EoE) is a chronic, local immune-mediated inflammatory oesophageal disease. Although Budesonide is recommended as one of the first-line drugs for EoE treatment, its efficacy is still controversial in multiple studies. Due to the continuous emergence of new and reliable research evidence in recent years, we updated the meta-analysis using RCT trial results to evaluate the efficacy and safety of budesonide.

**Materials and method:**

Retrieve the data of the randomised controlled trial literature from 2000 to June 20, 2021, on using Budesonide in the treatment of eosinophilic esophagitis from the three major databases. Based on the results achieved with the Cochrane risk assessment tool, evaluate the quality of the included literature to extract the data, and perform the Meta-analysis with RevMan5.4 and Stata15.0.

**Results:**

A total of 958 articles were retrieved, with 10 articles finally included, thus forming a sample size of 712 cases. The main outcome indicators of the meta-analysis are as follows: (1) Histological remission: the Budesonide group performs better than the placebo control group when it comes to histological remission of injuries [RR = 23.82, 95%CI = (13.46, 42.21), *p* < .001]; (2) Eosinophil count: the Budesonide group is superior to the control group in terms of reduced eosinophil count [SMD = −1.34, 95%CI = (−1.52, −1.15), *p* < .001].

**Conclusion:**

More and more high-quality randomised controlled trials show that oral budesonide in the treatment of eosinophils esophagitis was better than the placebo group. Mounting high-quality RCTs have confirmed the efficacy of oral budesonide in the treatment of eosinophilic esophagitis and that the effects of this drug may not be so dose-dependent. It is safe to take budesonide for a long time, and this drug is a relatively ideal option for drug treatment of eosinophilic esophagitis at present, so it is worthy of clinical application.

**Key Messages**
1. We used high-quality randomised controlled trials to meta-update the previous results to further confirm the clinical efficacy and safety of budesonide.
2. Oral budesonide in the treatment of eosinophilic esophagitis is significantly better than the placebo control group. We have confirmed the value of its clinical application and promotion by including more high-quality randomised controlled trials.
3. We also found that the efficacy of budesonide in patients is not dose-dependent, and more research is needed to confirm this.

## 1. Introduction

Eosinophilic esophagitis (EoE) is a chronic, local immune-mediated inflammatory oesophageal disease, which is defined in clinical practices as symptoms of oesophageal dysfunction and defined by histology as eosinophils-dominant oesophageal mucosal infiltration [1]. In the past few decades, the prevalence of EoE has been increasing dramatically [2,3]. The disease can be prevalent in all age groups and mostly affect males, and the ages of adult patients are usually 30–40 years old [4]. There may be differences in clinical characteristics of patients of different ages. In the case of infants and children, EoE may cause feeding problems, vomiting, heartburn, or abdominal pains, while in terms of adolescents and adults, the main symptoms are dysphagia and swallow-induced pains [5,6]. The life quality of EoE patients is affected by the severity levels of EoE disease accordingly, not only because of the symptom-induced burdens, but also owing to the difficulties they encounter in terms of their diets and social habits, and those difficulties caused by their lack of the knowledge of the direct causes of their symptoms [7]. In most cases, the natural course of the disease is progressive. Without active intervention and treatment, it may lead to oesophageal remodelling and a long-term high incidence of oesophageal stenosis [8,9].

Effective treatment of EoE is essential for controlling persistent symptoms, improving life quality, and decreasing the incidence of disease progression. At present, common treatments for EoE include drug therapy, diet therapy, and oesophageal dilatation [10]. Specifically, drugs such as budesonide, fluticasone propionate, proton pump inhibitor (PPI), and biological agents can be used for drug therapy. Among these drugs, some studies have confirmed [11–13] that budesonide, as a relatively ideal option for drug treatment of eosinophilic esophagitis, can inhibit oesophageal epithelial inflammation, repair the mucosal barrier, and promote tissue remodelling, to reduce the incidence of oesophageal stenosis and food impaction. However, there is still no expert consensus on the standardised application of budesonide in EOE treatment, due to the differences in the population of included studies, administration method, dose, cure, etc. A 2015 meta-analysis based on trials which are including but not limited to randomised controlled trials (RCTs) showed that steroids can effectively reduce the eosinophil count of EoE patients, and their values in improving symptoms have not been determined [14]. At the same time, a 2016 meta-analysis report based on randomised controlled trials (RCTs) showed that corticosteroids seem to have an effect in relieving EoE in terms of histology, but no similar effect is found in relieving clinical symptoms [4]. However, as previous similar meta-analyses included few studies with low quality, many high-quality randomised controlled trials have been published recently. Therefore, combined with a small number of early RCT results, it is of great help to evaluate the effectiveness and safety of BOS application in EOE.

## 2. Materials and methods

### 2.1. Study selection and data collection

#### 2.1.1. Data and methodology

#### 2.1.2. Objectives

Publicly published RCT literature on treating eosinophilic esophagitis with Budesonide in the 3 big databases of medical journals, namely, PubMed, Embase, and Cochrane Library (grey literature excluded).

### 2.2. Inclusion & exclusion criteria for literature

Inclusion criteria: (1) literature that reported treating EOE of the intervention group with Budesonide and using placebo in the control group; (2) articles reporting human data; (3) articles limited to the English language; (4) no restrictions on gender, age and Regional.

Exclusion criteria: (1) case reports, case series, letters, reviews and review articles; (2) data unable to be extracted; (3) overlapping data sets; (4) pure abstracts of papers; (5) animal or *in vitro* studies; (6) duplicate and irrelevant data.

### 2.3. Data sources and retrieval strategies

Two reviewers conducted independent literature searches in the three major databases, namely PubMed, Embase, and Cochrane Library, with the search time from 2000 to April 20, 2021. The following comprehensive search terms were used to collect all related articles: (Budesonide) AND (Eosinophilic esophagitis OR eosinophilic oesophagitis OR allergic oesophagitis). MeSH (Medical Subject Headings) terms used were “Budesonide” and “Eosinophilic Esophagitis.”

### 2.4. Data, related information extraction, and quality evaluation

Two independent researchers participated in the entire process of the literature search. The first round of screening was performed based on the title and the abstract to exclude research on irrelevant topics. Then, the included articles were screened according to the full text, with the unqualified articles inconsistent with the inclusion criteria excluded. Data extraction was performed by using a standardised data collection table. The extracted content included: document title, author, year, country, disease name, sample size, patient age, gender, gender ratio, intervention facilities of treatment group, intervention facilities of control, and drug administration route, course of the disease, course of treatment/follow-up time, histological diagnostic criteria. Literature screening, data extraction, and quality assessment were conducted independently by two authors. Any divergences were resolved *via* discussion until a consensus was reached. In the case of remaining disagreements, another researcher was invited to participate in the discussion to obtain final agreements. RCT literature quality evaluation was conducted by Cochrane Handbook 5.1.0 to evaluate the risk of bias tool of RCT. The evaluation content included the following aspects: ①random sequence generation; ② allocation concealment; ③Whether blinding would be implemented on subjects and lab personnel; ④ implementing blind test on effect index evaluation; ⑤ incomplete data of results; ⑥ selective reporting results; ⑦ bias from other sources.

### 2.5. Statistical processing

Meta-analysis of the data was conducted with RevMan5.4 and Stata15.0. Continuous variables were expressed by the standardised mean difference method (MD, SMD) and binary variables were expressed by relative risk (RR) and its 95% confidence intervals (CI). The heterogeneity test was performed among the studies. When *p* ≥ .1 and *I*^2^ < 50%, the heterogeneity was considered to be low, and the fixed effects model was adopted. When *p* < .1 and *I*^2^ > 50%, the heterogeneity was considered to be in existence, and Subgroup analysis and sensitivity analysis were adopted to explore the source of heterogeneity. When the source of heterogeneity was unable to be determined, a meta-analysis of the literature was conducted with a random effects model. The publication bias included in the study was expressed with Begg’s test, Egger’s test, and funnel plots. Meta-analysis of Begg’s test and Egger’s test *p* < .05 showed the statistical difference.

## 3. Results

### 3.1. Literature search results

**Figure 1.**
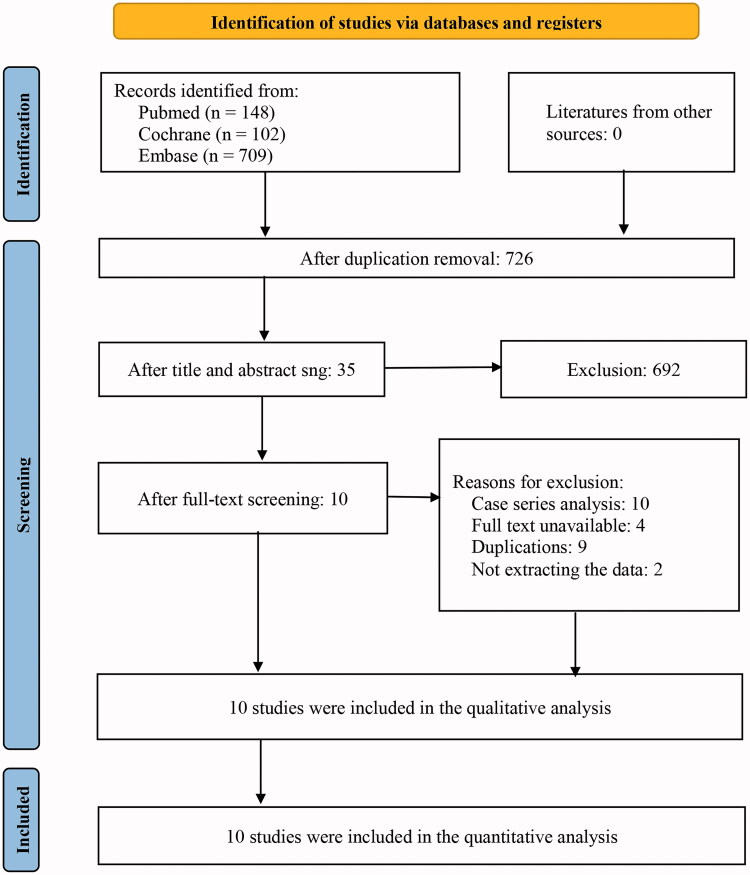
Flowchart of selection and screening of the studies.

Preliminary screening of 959 relevant literature was carried out, with 10 randomised controlled studies finally included after progressively repeated screening [15–24] for Meta-analysis. Totally 955 patients were included in the study, with 544 in the Budesonide group (experimental group), and 411 in the placebo group (control group). The literature screening flowchart is shown in Figure 1.

### 3.2. Basic characteristics of included literature

Included in the study were all randomised controlled experimental studies published from 2000 to June 20, 2021 [15–24], involving 955 patients with an average age between 7.8 and 46.5. Participants were administered oral Budesonide, with the treatment course ranging from 2 weeks to 48 weeks. Table 1 shows the detailed data of the baseline characteristics of the participants included.

**Table 1.** Baseline characteristics.

First author/year/country	Sample size		Intervention/Route/dose		Average age (year)*		Duration of treatment (week)	Histologic diagnostic criteria:(eos/ hpf)
	I	C	I	C	I	C		
Straumann/2020/Germany [12]	68	68	Budesonide Oral/0.5 mg	Placebo	36 ± 10.9	36 ± 9.9	48	<15
	68	68	Budesonide Oral/1.0 mg	Placebo	37 ± 11.1	36 ± 9.9	48	<15
Lucendo/2019/Spain [13]	59	29	Budesonide Oral/1.0 mg	Placebo	37 ± 11.5	37 ± 9.2	6	>20
Dellon/2018/America [14]	45	37	Budesonide Oral/2.0 mg	Placebo	22.4 ± 7.8	21.2 ± 7.7	12	>15
Dellon/2016/America [15]	51	42	Budesonide Oral/2.0 mg	Placebo	22.3 ± 7.9	20.8 ± 7.5	4	>15
Schlag/2015/Germany [16]	51	18	Budesonide Oral/2.0 mg	Placebo	40.8 ± 13.8	36.2 ± 10.2	2	/
Gupta/2015/ America [17]	21	21	Budesonide Oral/0.5 mg	Placebo	9 ± 5.88	9.2 ± 4.36	12	>20
	21	21	Budesonide Oral/2.0 mg	Placebo	10.2 ± 4.89	9.2 ± 4.36	12	>20
	21	21	Budesonide Oral/4.0 mg	Placebo	8.1 ± 4.58	9.2 ± 4.36	12	>20
Miehlke/2015/Germany [18]	19	19	Budesonide Oral/1.0 mg	Placebo	38.9 ± 12.6	16 ± 84.2	2	≥20
	19	19	Budesonide Oral/2.0 mg	Placebo	37.2 ± 13.9	16 ± 84.2	2	/
	19	19	Budesonide Oral/5.0 ml	Placebo	46.5 ± 14.1	16 ± 84.2	2	/
Strumann/2011/Switzerland [19]	14	14	Budesonide Oral/2.0 mg	Placebo	38.0 ± 11.7	34.0 ± 13.9	2	/
Dohil/2010/ America [20]	15	9	Budesonide Oral/1.0 mg or 2.0 mg	Placebo	7.8 ± 4.8	7.7 ± 4.8	9	≥20
Straumann/2010/Switzerland [21]	18	18	Budesonide Oral/1.0 mg	Placebo	33.1 ± 13.1	38.2 ± 12.4	2	≥20

I = intervention; C = control; *mean ± sd; eos/hpf eosinophils per high-powered field.

### 3.3. Quality evaluation of literature included

**Figure 2.**
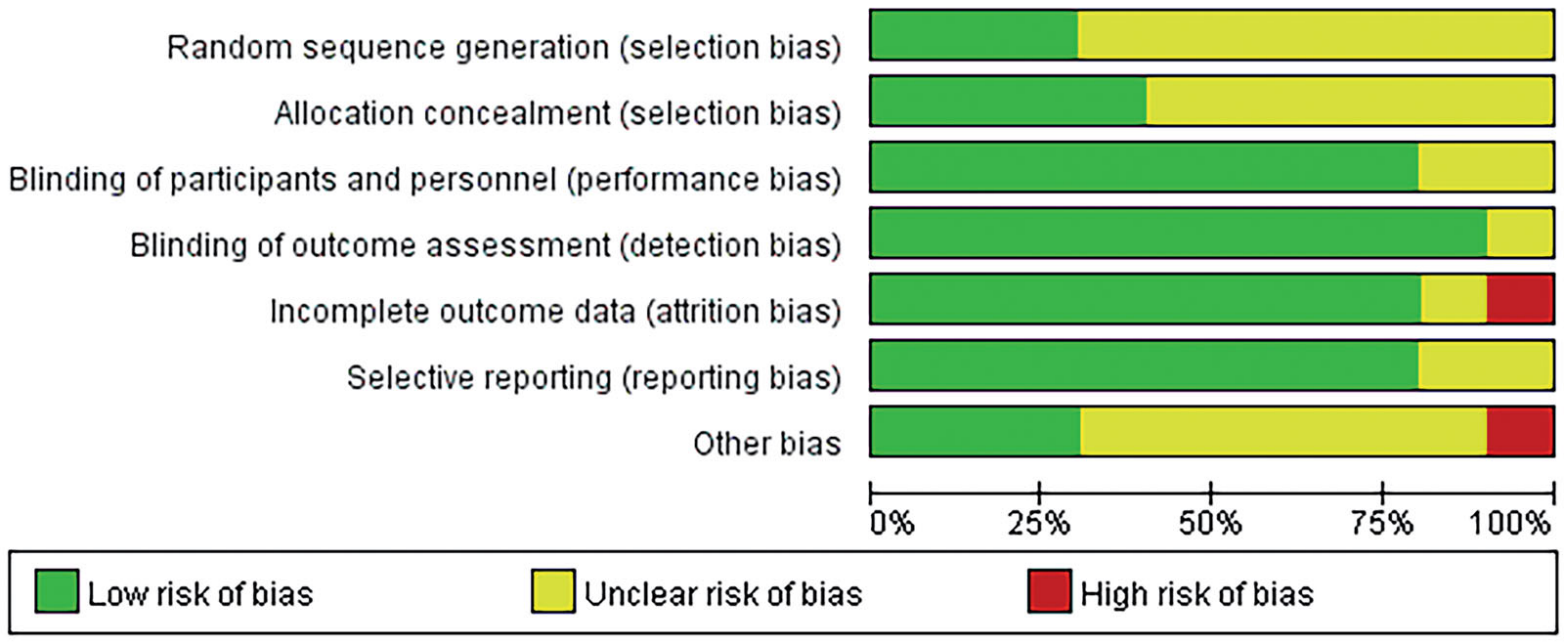
Risk of bias graph.

**Figure 3.**
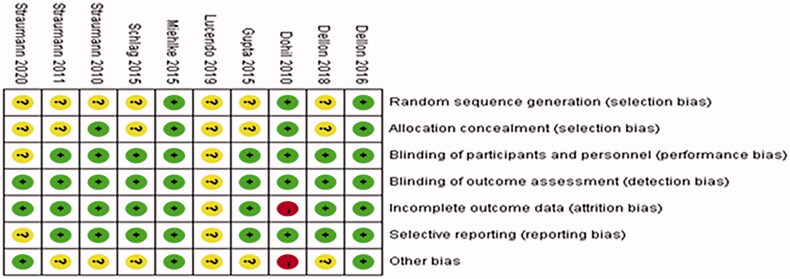
Risk of bias summary.

The literature included in the study was evaluated with Revman5.3 to label the risk of literature bias as “low risk,” “high risk” and “unclear risk,” while the evaluation items included random sequence generation, allocation concealment, blinding, and outcome indicators, as shown in Figures 2 and 3.

### 3.4. Meta-analysis

#### 3.4.1. Histological remission

**Figure 4.**
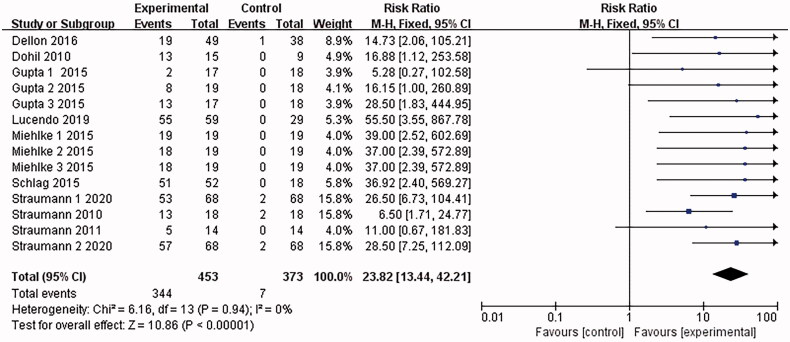
Histological remission forest plot. Note: Different serial numbers represent different specifications of Budesonide.

10 papers [15–24] reported histological remission. *I*^2^ = 85% was found after the heterogeneity test. The random effects model was adopted. Then the sensitivity analysis was conducted with the findings that the results were largely affected by Dellon 2018, which was excluded. 9 documents remained after the exclusion, and the combined results were not heterogeneous (*I*^2^ = 0%). The analysis was conducted with the fixed-effect model, which led to the belief that the eosinophilic esophagitis group treated with Budesonide performed better than the placebo control group in terms of histological remission [RR = 23.82, 95%CI = (13.46, 42.21), *p* < .00001] The difference was statistically significant (as shown in Figure 4).

#### 3.4.2. Eosinophilic count

**Figure 5.**
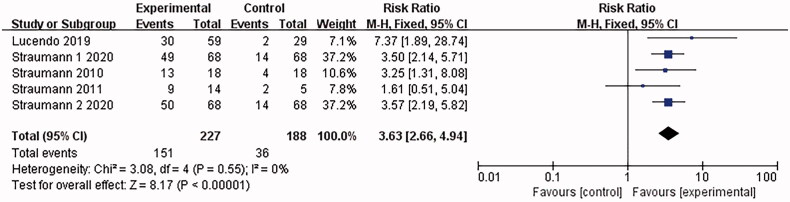
Eosinophilic count forest plot. Note: Different serial numbers represent different specifications of Budesonide.

8 papers [15–19,22–24] reported the eosinophil count. *I*^2^ = 80% was found after the heterogeneity test. The random effects model was adopted. Then the sensitivity analysis was conducted with the findings that the results were largely affected by Dellon 2018, which was excluded. 7 documents remained after the exclusion, and the combined results showed no significant heterogeneity (*I*^2^ = 1%). The analysis was conducted with the fixed-effect model, which led to the belief that the eosinophilic esophagitis group treated with Budesonide performed better than the placebo control group in reducing eosinophils [SMD = −1.34, 95%CI = (−1.52, −1.15), *p* < .00001]. the difference was statistically significant (as shown in Figure 5).

#### 3.4.3. Clinical response

**Figure 6.**
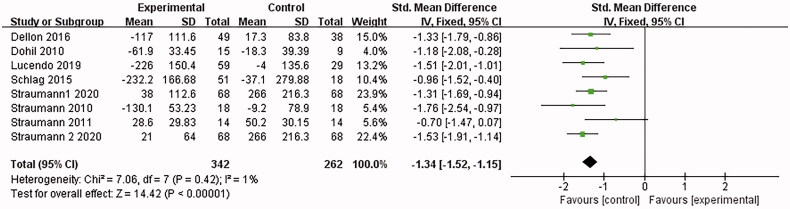
Clinical response forest plot. Note: Different serial numbers represent different specifications of Budesonide.

4 articles [15,16,22,24] involved clinical response, and no heterogeneity was shown in the combined results (*I*^2^ = 0%). The analysis was conducted with the fixed-effect model, which led to the belief that the eosinophilic esophagitis group treated with Budesonide gained significant improvement in clinical symptoms than the placebo control group [RR = 3.63, 95%CI = (2.66, 4.94), *p* < .00001]. The difference was statistically significant (as shown in Figure 6).

#### 3.4.4. Endoscopic changes

**Figure 7.**
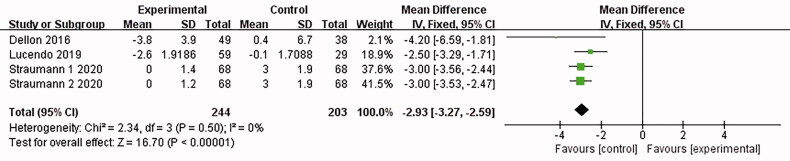
Endoscopic changes forest plot. Note: Different serial numbers represent different specifications of Budesonide.

3 articles [15,16,18] reported relevant abnormal endoscopic changes (wrinkles, rings, edoema, structure, and leukoplakia/plaque/exudation), and no heterogeneity was shown in the combined results (*I*^2^ = 0%). The analysis was conducted with the fixed-effect model, which led to the belief that compared with the placebo control group, the eosinophilic esophagitis group treated with Budesonide showed a significant decrease in endoscopy [MD = −2.93, 95%CI = (−3.27, −2.59), *p* < .00001]. The difference was statistically significant (as shown in Figure 7).

#### 3.4.5. Symptoms

**Figure 8.**
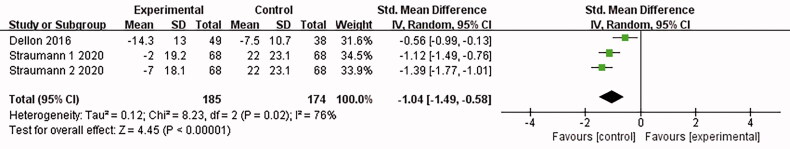
Symptoms forest plot. Note: Different serial numbers represent different specifications of Budesonide.

Two papers [15,18] reported symptom scores, and the combined results were highly heterogeneous (*I*^2^ = 76%). The analysis was conducted with the fixed-effect model, which led to the belief that adverse symptoms of the patients treated with Budesonide were better alleviated than those of the placebo control group (swallow-induced pains and dysphagia) [SMD = −1.04, 95%CI = (−1.49, −0.58), *p* < .00001]. The difference was statistically significant (as shown in Figure 8).

#### 3.4.6. Incidence of drug-related adverse events

**Figure 9.**
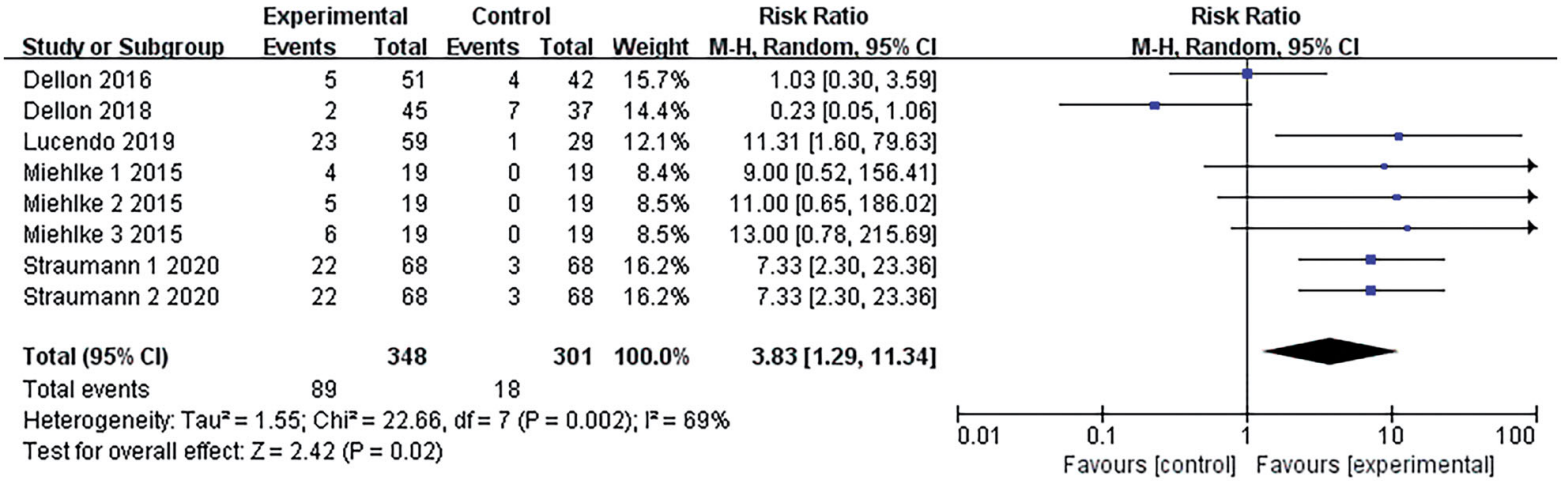
Forest plot of incidence of drug-related adverse events. Note: Different serial numbers represent different specifications of Budesonide.

5 articles [15–18,21] reported adverse drug events (mostly oral and oesophageal Candida infections, and a few nausea and dizziness), and the combined results were highly heterogeneous (*I*^2^ = 69%). The analysis was conducted with the random-effects model, which led to the belief that compared with the placebo control group, patients treated with Budesonide were more likely to have adverse drug events [RR = 3.83, 95%CI = (1.29, 11.34), *p* = .02]. The difference is statistically significant. By the sensitivity analysis of deleting every single study one by one, Dellon 2018 was found to be the main source of heterogeneity (as shown in Figure 9).

### 3.5. Publication bias

**Figure 10.**
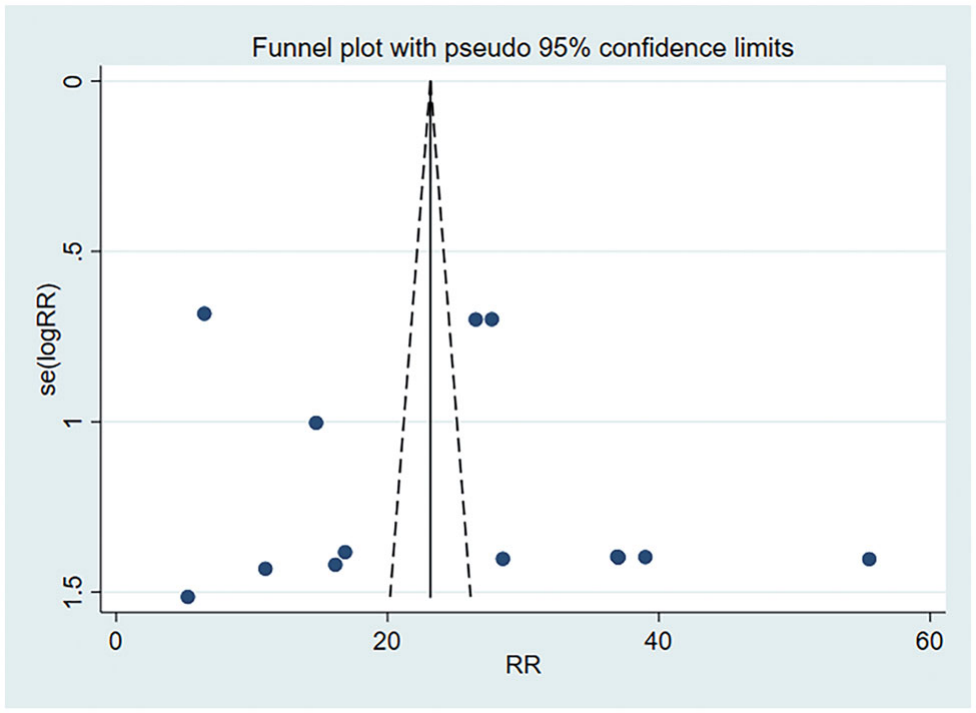
Histological remission funnel plot.

**Figure 11.**
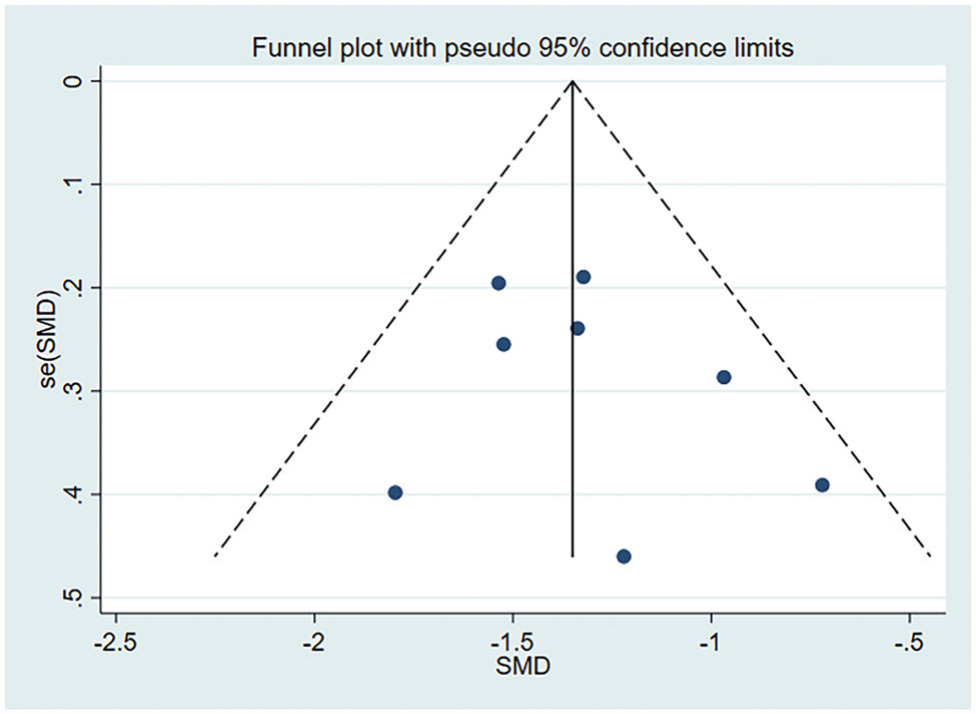
Eosinophilic count funnel plot.

A funnel plot involving the included studies on histological remission and eosinophil count was drawn with Stata15.0, and Begg’s test and Egger’s test were conducted to quantify the funnel plot. The funnel plot was substantially symmetrical, suggesting less possibility of publication bias. Begg’s test and Egger’s test: histological remission rate by Begg’s Test was *p* = .956 and that by Egger’s Test was *p* = .277, suggesting no publication bias. Eosinophil count by Begg’s was Test *p* = .711 and that by Egger’s Test was *p* = .464, suggesting no publication bias (as shown in Figures 10 and 11).

## 4. Discussion

In this study, the histological relief of injury in the budesonide group was superior to that in the placebo group. The decrease of eosinophil count in the budesonide group was better than that in the control group. Compared with the placebo group, the clinical symptoms of the budesonide group were significantly improved; There were significantly fewer endoscopic abnormalities in the budesonide group compared with the placebo group; Adverse symptoms were significantly reduced in the budesonide group compared with the placebo. Patients in the budesonide group were more likely to have drug-related adverse events than those in the placebo group, consistent with the findings of previous meta-analyses [25]. But we are in the study also found abnormal under endoscope and symptom scores were improved, this is the institute made no mention of before, the more can prove budesonide for Eosinophilic Esophagitis is effective.

Immune-mediated inflammatory disease is characterised by various inflammatory cells participating in the occurrence and development of allergic reactions. The pathologic changes in eosinophil (Eos) infiltration are an important indicator to judge the degree of inflammatory reaction, and diagnosis and treatment of allergic diseases [26]. From the study results, peripheral blood eosinophil counts in the budesonide group were significantly lower than those in the placebo group. Eosinophil density is the most important local disease activity marker of EoE [15] and is often used as one of the main endpoints of EoE treatment. The significant reduction of eosinophils in patients with EoE is a marker of histological remission, suggesting that budesonide may be involved in the pathogenesis of EoE, which can effectively reduce the inflammatory mediators of the oesophageal mucosa and reduce the histological response of PATIENTS with EoE. However, eosinophils should be used cautiously to evaluate their efficacy in patients with EoE for the following reasons: first, eosinophils are susceptible to confounding factors, such as seasonal factors and specific diseases; Secondly, so far, few studies have systematically evaluated the therapeutic value of blood eosinophils monitoring EoE [27,28], and the results are controversial. Third, studies have shown that only post-treatment peripheral blood eosinophil/mm3 values ≤300 can reliably predict histological remission.

The attention should not only be paid to the remission of histological response, but also to the improvement of clinical symptoms (swallowing difficulties and swallow-induced pains) when it comes to the evaluation indexes of the EoE treatment effect. The results of this study showed that the symptom score of the Budesonide group was significantly reduced compared with that of the placebo group. However, our study results are only statistically significant. Whether there is a clinically significant improvement is still uncertain. This can only be finalised through the actual observation of the improvement in the patient's clinical symptoms. In addition, from this study, the endoscopic abnormalities (wrinkles, rings, edoema, structure, and leukoplakia/plaque/exudation) of the Budesonide group were also significantly improved compared with those of the placebo group, which indicates that Budesonide may significantly alleviate EoE patients’ adverse clinical symptoms, such as swallow-induced pains and dysphagia, thereby improving the quality of patients’ life.

From this study, although oral Budesonide was found to have a better effect in the treatment of EoE, a higher risk of drug-related adverse events was found in the Budesonide treatment group than that in the placebo group, and most of the adverse events were oral and oesophageal Candida infections, and a few nausea and dizziness in a few patients. Adverse reactions were speculated to be related to the dosage. Studies are reporting [15,21] that there is no significant difference in the risk of adverse reactions in the case of dosage of 0.5–1 m. When the dosage exceeds 1 mg, the risk of adverse reactions will increase as the dosage increases. Adverse reactions to budesonide, the most common fungal infection, can be easily treated with antimicrobial therapy and are effective, so there is no need to stop the study. The study showed that budesonide is safer for long-term use. Straumannetal [15] conducted a 48-week multi-center phase III clinical trial to study the effectiveness and safety of long-term use of BOT. In his study, most of the adverse events were minor, and no serious drug-related adverse events occurred. The incidence of local Candidiasis in the budesonide group was higher, but it was easy to treat and hardly interfered with the patients’ daily life activities. However, they reported that four patients had decreased serum cortisol levels under BOT without adrenal insufficiency. Therefore, we recommend that the symptoms and signs of adrenal insufficiency should be monitored in patients who are treated with BOT for more than 48 weeks or longer, especially in children and in those at higher doses.

In addition, statistics were also made in this study on the correlation between the different dosages and the efficacy as well as adverse reactions. The dosages included in the original study were 0.5, 1, 2, 4, 5 mg, etc., and there was no significant difference in the outcome effects of different dosages. This suggested that the efficacy of BOS in the treatment of EoE has nothing to do with the dosage levels and high-dose medications may not help achieve better efficacy than low-dose ones, but high-dose medications may increase the incidence of adverse reactions and other potential risks. Therefore, for the treatment of EoE, the recommended oral dose of BOS is 0.5 mg or 1.0 mg twice a day. There are studies showing that these two dosages are effective in reducing symptoms, maintaining clinical histological remission, and inhibiting inflammation. It has been reported [15] that for ordinary adult EoE patients, 0.5 mg twice a day may be sufficient to successfully maintain long-term remission. There are also meta-analyses indicating that 1 mg of oral Budesonide dispersible tablets twice a day is the best dosage for treating EoE [29]. In general, there is still a lack of investigations into the best dosage of BOS. It is expected that more studies can be carried out in the future to further determine the best dosage of BOS for the maintenance treatment of EoE.

The drug therapy for EOE mainly involves drugs, dietotherapy, oesophageal dilation, and lifestyle adjustment, which can be summarised as “3D.” Dietotherapy aims to avoid food-derived antigen stimulation [30,31]. However, due to the poor compliance of patients, there is still controversy about the long-term efficacy of drug treatments. Moreover, IgE-mediated food allergy may be induced after applying the elimination diet for the first time [32], and a long-term elimination diet may also cause malnutrition in patients. PPI can reduce the damage of acid substances to the oesophagus and block the Th2 immune response by inhibiting gastric acid secretion, to play a protective role against the oesophageal epithelial barrier damaged by the exposure of inflammatory factors [33]. Although PPI is currently widely recognised as the first-line drug for the treatment of EOE, it cannot be used as the first choice for long-term treatment, and it is not suitable for patients with narrow-caliber oesophagus or severe symptoms of EOE. At present, biological agents such as monoclonal antibodies are a new choice for EOE treatment, and they are relatively safe in clinical application, but the evidence of side effects and efficacy is still insufficient. In addition, fluticasone propionate and budesonide are both effective in reducing eosinophilia [34]. However, a systematic review by Murali AR *et al.* [4] showed that budesonide was significantly better than fluticasone propionate in terms of relieving symptoms. All in all, budesonide is still the first choice for the treatment of eosinophilic esophagitis at present because it is effective and relatively safe.

There are the following limitations in this study: (1) The small number of included studies and the lack of subgroups for adults and children to discuss may limit the stability and generalisation of results. (2) Limited by the number of included studies, dose-response studies were not carried out; (3) Some outcome indicators showed high heterogeneity, which may affect the results; (4) Due to the different doses of budesonide in each included literature, the heterogeneity of the articles would also be increased.

## 5 Conclusions

In conclusion, our research results showed that budesonide in the treatment of eosinophilic esophagitis can effectively reduce the number of eosinophils, improve the histological response, alleviate clinical symptoms, and endoscopic abnormalities, and has good long-term safety. Because several RCT studies have shown that the efficacy of budesonide on EOE is not dose-dependent, 0.5–1.0 mg twice daily oral administration can be used as the recommended dose of budesonide in the treatment of EOE. This can provide a reference for clinicians to choose the oral dose of budesonide. Due to the limitations of this study, more high-quality RCTs with large sample sizes can be carried out in the future to further clarify the above conclusions and actively look for the best dose of budesonide and the most suitable population.

## Data Availability

The data in the study are available from the corresponding author on reasonable request.
